# A Miniature Inductive Encoder for Linear Displacement Measurement

**DOI:** 10.3390/s26030973

**Published:** 2026-02-02

**Authors:** Wei Xiong, Shouhao Wang, Yajun Ma, Peng Chen, Sijia Cao, Jiajia Xu, Yanxu Wang

**Affiliations:** 1 Beijing Institute of Precise Mechanical and Electronic Control Equipment, Beijing 100076, China; xiongwei99@163.com (W.X.);; 2 Innovation Center for Control Actuators, Beijing 100076, China

**Keywords:** inductive encoder, linear displacement, eddy current, planar coils

## Abstract

In order to satisfy the measurement of objects in compact settings, a miniaturized linear inductive encoder with a measurement range of 15 mm is investigated in this paper. The encoder structure integrates a movable part with conductive plates and a stationary part with planar excitation and inductive coils. When a high-frequency alternating current is applied to the excitation coils, a time-varying magnetic field will be generated. Meanwhile, the conductive plates on the movable element will produce an eddy current magnetic field to reduce or boost the magnetic field. As the movable part moves, two-channel amplitude-modulated electrical signals whose amplitudes vary with displacement are obtained. The CORDIC algorithm is utilized to calculate the displacement. The paper describes the structure and working principle of the encoder, presents corresponding finite element simulations of the magnetic field, and introduces a prototype fabricated by PCB technology. Experiments evaluating stability, resolution, and accuracy show that the encoder reaches the measurement accuracy of 12.8 μm within one pitch, and the resolution is 0.7 μm. Importantly, its minimal dimensions (20 mm × 10 mm × 1 mm) enable installation in highly constrained mechanisms.

## 1. Introduction

By delivering precise positional feedback, the linear displacement encoder enables accurate motion control in linear motors, ball screw drives, or other linear motion systems. In parallel, encoder miniaturization has become an active research area to meet the demands of compact electronic devices, biotechnology, and advanced materials [1,2,3,4,5]. Distinguished by measurement principle, displacement encoders are classically categorized by their measurement principle, with the main types being optical, capacitive, and inductive encoders.

Optical displacement encoders have become indispensable in precision measurement applications due to their exceptional accuracy, measurement stability, and sensitivity [6,7,8]. These encoders operate on the fundamental principle of spatial light modulation through precisely fabricated grating structures with equally spaced rulings [9]. Displacement measurement is achieved by photoelectric detection and subsequent counting of grating lines, which necessitates extremely high manufacturing precision and geometric consistency in the grating patterns. Recent advancements in optical encoder technology have focused on enhancing resolution through innovative signal processing techniques. Renishaw’s stabilized interferometer achieves a worst-case linear error of ±0.5 ppm and a resolution of 1 nm through precision-stabilized laser sources and advanced environmental compensation [10]. Notably, Wang developed a high-precision peak localization method utilizing grating image analysis, achieving nanometer-level resolution with submicron accuracy [11]. This approach incorporates local gradient interpolation algorithms to optimize displacement curve fitting, demonstrating a local measurement accuracy of ±0.1 μm and a full-range accuracy of ±1 μm over the 50 mm travel range. While many studies have introduced error compensation for optical linear encoders, their performance is still constrained by the diffraction barrier of ultra-precision engraving and optical scanning [12].

Capacitive displacement encoders are widely used in nanopositioning platforms due to their excellent resolution and low cost [13]. The working principle relies on motion-induced capacitance variation between parallel electrodes maintained at a constant separation [14,15,16]. To address the inherent range limitations of capacitive displacement encoders, researchers have developed various technical approaches for enhancing the measurement range. For example, Peng [16] employed a time-pulse counting method to measure displacement, thereby optimizing the performance of capacitive encoders and achieving an improved accuracy of ±200 nm. The proposed method in [17] achieves 0.9 nm resolution within a measurement range of 15 mm and linearity error below 0.0026% of full scale, by implementing periodic capacitance variation detection combined with diamond-like carbon (DLC) film electrodes. Due to stray capacitance and edge effects, capacitive displacement encoders exhibit nonlinear characteristics, and the dielectric constant between the two plates is sensitive to environmental variables such as temperature and humidity [18].

Inductive displacement encoders are widely used in machining, military testing, product quality testing, and other industrial production fields [18,19,20]. Based on the principle of electromagnetic induction effect, eddy current encoders realize displacement measurement through the mutual inductance variation between the coil and the metal conductor. The study in [21] achieved a unique resolution of 6.7 nm over a 5 mm measurement range through the compensation of coil inductance and capacitance tolerances. Tong proposed a novel high-precision, long-range eddy current encoder that achieves a measurement accuracy of 6.75 μm over a 500 mm travel range [22]. Some other eddy current encoders can achieve ±20 μm accuracy in a range of 300 mm [23]. A planar coil pair with an E-type soft ferromagnetic core was adopted in [24], achieving a resolution of 2 µm over a range of 70 mm. In [25], a Y-type core structure was used, attaining a resolution of 6.5 µm within 86 mm. By further extending the range, Gu [26] implemented a bilateral sensing units structure, which reached an accuracy of 15 µm and a resolution of 7 µm over a span of 288 mm. Linear displacement encoders based on electromagnetic induction are widely used in industrial production due to their reliability, immunity to environmental interference, high accuracy, and cost-effectiveness.

This paper presents a compact linear displacement encoder based on electromagnetic induction principles. In contrast to conventional eddy current encoders, the proposed design achieves a substantial reduction in physical footprint without compromising the measurement range, rendering it ideal for space-constrained applications. Despite its minimized size, the encoder retains high precision and demonstrates sufficient stability for reliable operation in harsh environmental conditions. The paper is structured as follows: Section 2 introduces the encoder’s structure, working principle, mathematical model, and simulation results. Section 3 provides a detailed analysis of these simulations. Experimental validation and a comprehensive performance evaluation are presented in Section 4. Finally, Section 5 concludes the paper by summarizing the key findings and suggesting potential directions for future work.

## 2. Structure and Measurement Principle

### 2.1. Structure and Electromagnetic Induction

**Figure 1:**
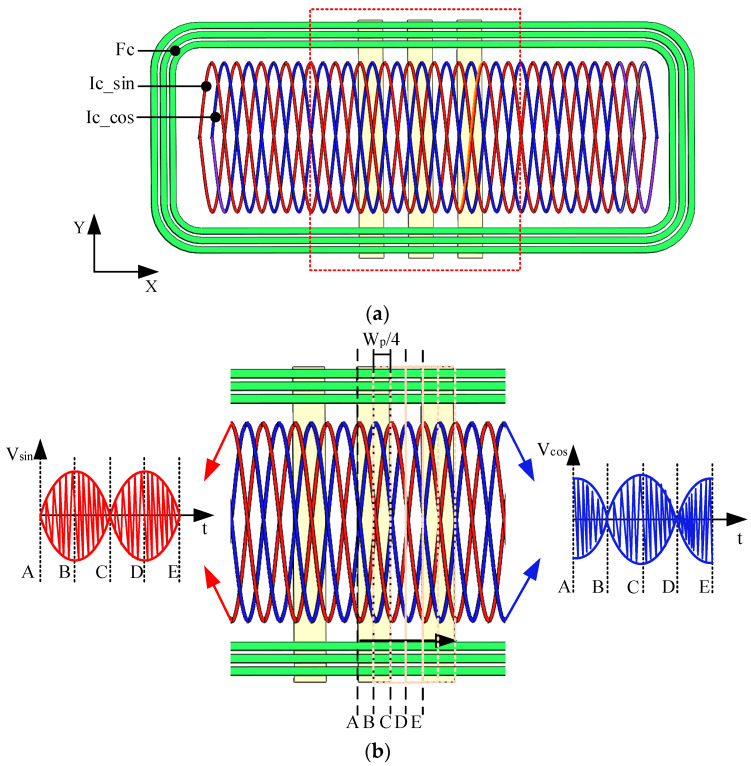
Encoder structure: (**a**) simplified diagram of the encoder structure; (**b**) detail view of the encoder and inductive signals of Ic_Sin(V_sin_) and Ic_Cos(V_cos_).

The structure of the miniaturized inductive linear displacement encoder is depicted in Figure 1a. It comprises a movable part and a stationary part. The movable part incorporates three metal conductive plates, while the stationary part houses the excitation and inductive coils. The excitation coils, designated as Fc, are a rectangular helical coil aligned with the measurement direction (x-axis). Two inductive coils, Ic_sin and Ic_cos, are internally distributed in a staggered configuration with a spatial offset of W_p_/4, where W_p_ denotes the encoder pitch.

A partial view of the encoder is shown in Figure 1b. The excitation coil (Fc) is illustrated by the green curve. The inductive coils are represented by the red (Ic_sin) and blue (Ic_cos) curves. The dashed lines indicate the positions of Ic_sin and Ic_cos after a displacement of one pitch. The metal conductive plates have a width of W_p_/2 and a length exceeding the width of Fc.

The excitation coils are supplied with an alternating current (AC) signal *I* = *I*_0_sin (*ωt*), where *I*_0_ is the amplitude, *ω* is the angular frequency of the excitation signal. Based on the second-order vector potential theory, the time-varying magnetic field produced by the excitation signal in the surrounding space is analytically solved [27,28]. The magnetic field *B_s_* can be expressed as follows:

(1) $$B_{s}=\nabla \times A=\nabla \times (\nabla \times W)$$

where ∇ is the gradient operator, *A* is the vector magnetic potential, and *W* is the second-order vector potential. *W* can be decomposed into two orthogonal components. Selecting the z-axis unit vector *e_z_* in the Cartesian coordinate system for decomposition, the decomposed *W* can be expressed as follows:

(2) $$W=W_{a}\;e_{z}\;+\nabla \times (W_{b}\;e_{z\;})$$

Here, *W_a_* and *W_b_* are both scalar potential functions. Introducing the Coulomb gauge ∇ *A* = 0, the analytical solution for the vector magnetic potential *A* is obtained by solving the vector Helmholtz equation.

(3) $$\nabla^{2}A+\gamma^{2}A=0,\gamma^{2}=-j\omega \mu \sigma$$

where *γ*^2^ is the wavenumber and *γ*^2^ = −*jωμσ*, *ω* denotes the angular frequency of the excitation signal, *μ* is the magnetic permeability of the medium, and *σ* is the electrical conductivity of the medium. Since the coil is encased in an air medium, *γ*^2^ = 0. Combining Equations (1)–(3), the time-varying magnetic field generated by excitation coils is expressed as follows:

(4) $$\begin{array}{cc} B_{s} & =\nabla (\frac{\partial W}{\partial z})=\frac{\mu_{0}I}{2\pi^{2}(z_{b}-z_{a})w}\sum_{m=1}^{M}\sum_{n=1}^{N}P_{N}\left(e^{-kz_{bM}}-e^{-kz_{aM}}\right)\\ & \times \int_{-\infty }^{+\infty }\int_{-\infty }^{+\infty }\left[\frac{1}{-k}\left(e^{kz}-\frac{k\mu_{r}-\lambda }{k\mu_{r}+\lambda }e^{-kz}\right)\left(jme_{x}+jne_{y}\right)-\left(e^{kz}+\frac{k\mu_{r}-\lambda }{k\mu_{r}+\lambda }e^{-kz}\right)e_{z}\right]\\ & \times \frac{1}{mn}e^{jmx}e^{jny}dmdn \end{array}$$

(5) $$P_{N}=\left\{\begin{array}{l} \frac{\sin\left[\left(m-n\right)w+mx_{0N}-ny_{0N}\right]-\sin\left(mx_{0N}-ny_{0N}\right)}{2\left(m-n\right)}\\ -\frac{\sin\left[\left(m+n\right)w+mx_{0N}+ny_{0N}\right]-\sin\left(mx_{0N}+ny_{0N}\right)}{2\left(m+n\right)},m\neq n\\ \frac{w}{2}\cos\left[m\left(x_{0N}-y_{0N}\right)\right]-\frac{\sin\left[m\left(2w+x_{0N}+y_{0N}\right)\right]-\sin\left[m\left(x_{0N}+y_{0N}\right)\right]}{4m},m=n \end{array}\right.$$

where *μ*_0_ is the permeability of free space, *z* is the distance between excitation coils and metal conductive plates, *z_b_* and *z_a_* are the distances from the upper and lower end faces of the excitation coils to the metal conductive plate, *w* is the trace width of excitation coils, *x*_0_ and *y*_0_ are the distances from the excitation coils to the y-axis and the *x*-axis, *m* and *n* are integration variables, *k*^2^ = *m*^2^ + *n*^2^, *λ*^2^ = *k*^2^ + *γ*^2^. When a rectangular coil carrying a high-frequency alternating current is placed above a metal conductor, eddy currents J*_e_* are induced within the conductor. Meanwhile, the magnetic field generated by the eddy currents can be expressed as follows:

(6) $$\begin{array}{cc} J_{e} & =\frac{j\omega \sigma \mu_{0}I}{\pi^{2}w\left(z_{b}-z_{a}\right)}\frac{\mu_{r}e^{\lambda z}}{k\left(k\mu_{r}+\lambda \right)}\sum_{M=1}^{M}\sum_{N=1}^{N}P_{N}\left(e^{-kz_{bM}}-e^{-kz_{aM}}\right)\\ & \times \int_{-\infty }^{+\infty }\int_{-\infty }^{+\infty }\frac{1}{mn}\left(jme_{x}-jne_{y}\right)e^{jmx}e^{jny}dmdn \end{array}$$

(7) $$\begin{array}{cc} B_{e} & =\frac{j\omega \sigma \mu_{0}^{2}\mu_{r}I}{\pi^{2}w\left(z_{b}-z_{a}\right)}\frac{\mu_{r}e^{\lambda z}}{k\left(k\mu_{r}+\lambda \right)}\sum_{M=1}^{M}\sum_{N=1}^{N}P_{N}\left(e^{-kz_{bM}}-e^{-kz_{aM}}\right)\\ & \times \int_{-\infty }^{+\infty }\int_{-\infty }^{+\infty }\left(-\frac{e_{x}}{m^{2}}+\frac{e_{y}}{n^{2}}\right)e^{jmx}e^{jny}dmdn \end{array}$$

As shown in Figure 1a, the geometry of inductive coils can be expressed as Asin (2*πx*/*W_p_*), where A is the coils’ amplitude. Thus, the magnetic flux through the induction coils can be expressed as follows:

(8) $$\begin{array}{cc} \phi & =\frac{AW_{p}}{\pi }\cos\left(\frac{2\pi }{W_{p}}x\right)\left(B_{s}-{Β}_{e}\right)\\ & =\cos\left(\frac{2\pi }{W_{p}}x\right)\frac{j\omega \sigma \mu_{0}^{2}AW_{p}I}{\pi^{2}w\left(z_{b}-z_{a}\right)}\frac{\mu_{r}^{2}e^{\lambda z}}{k\left(k\mu_{r}+\lambda \right)}\sum_{M=1}^{M}\sum_{N=1}^{N}P_{N}\left(e^{-kz_{bM}}-e^{-kz_{aM}}\right)\\ & \times \int_{-\infty }^{+\infty }\int_{-\infty }^{+\infty }\left(-\frac{e_{x}}{m^{2}}+\frac{e_{y}}{n^{2}}\right)e^{j\left(mx+ny\right)}dmdn \end{array}$$

According to the law of electromagnetic induction, the induced electromotive force (EMF) of Ic_sin can be described as follows:

(9) $$\begin{array}{cc} U\left(t,x\right) & =-\frac{d\phi }{dt}=-\cos\left(\frac{2\pi }{W_{p}}x\right)\frac{\omega^{2}\sigma \mu_{0}^{2}AW_{p}I_{0}e^{j\omega t}}{\pi^{2}w\left(z_{b}-z_{a}\right)}\frac{\mu_{r}^{2}e^{\lambda z}}{k\left(k\mu_{r}+\lambda \right)}\sum_{M=1}^{M}\sum_{N=1}^{N}P_{N}\left(e^{-kz_{bM}}-e^{-kz_{aM}}\right)\\ & \times \int_{-\infty }^{+\infty }\int_{-\infty }^{+\infty }\left(-\frac{e_{x}}{m^{2}}+\frac{e_{y}}{n^{2}}\right)e^{jmx}e^{jny}dmdn \end{array}$$

The spatial separation between Ic_sin and Ic_cos is W_p_/4. By expanding the induced EMF in a Fourier series and taking only its real part, the EMFs of Ic_sin and Ic_cos can be expressed as follows:

(10) $$\left\{\begin{array}{cc} U_{1}\left(t,x\right) & =\cos\left(\frac{2\pi }{W_{p}}x\right)\sum_{m,n}f\left(m,n\right)\cos\left(mx+ny\right)\left(-\frac{e_{x}}{m^{2}}+\frac{e_{y}}{n^{2}}\right)\cos\left(\omega t\right)\\ U_{2}\left(t,x\right) & =\cos\left(\frac{2\pi }{W_{p}}\left(x+\frac{W_{p}}{4}\right)\right)\sum_{m,n}f\left(m,n\right)\cos\left(mx+ny\right)\left(-\frac{e_{x}}{m^{2}}+\frac{e_{y}}{n^{2}}\right)\cos\left(\omega t\right)\\ & =-\sin\left(\frac{2\pi }{W_{p}}x\right)\sum_{m,n}f\left(m,n\right)\cos\left(mx+ny\right)\left(-\frac{e_{x}}{m^{2}}+\frac{e_{y}}{n^{2}}\right)\cos\left(\omega t\right) \end{array}\right.$$

(11) $$f\left(m,n\right)=-\frac{\omega^{2}\sigma \mu_{0}^{2}AW_{p}I_{0}}{\pi^{2}w\left(z_{b}-z_{a}\right)}\frac{\mu_{r}^{2}e^{\lambda z}}{k\left(k\mu_{r}+\lambda \right)}\sum_{N=1}^{N}\sum_{M=1}^{M}P_{N}\left(e^{-kz_{bM}}-e^{-kz_{aM}}\right)$$

f(m,n) is mainly related to the excitation current, the material parameters σ and μ_r_, the coil’s geometry, size, and position, as well as the settings of the boundary conditions. By applying the arctangent function, the displacement is obtained.

(12) $$x_{1}=\frac{W_{p}}{2\pi }\arctan\left(\frac{U_{2}\left(t,x_{1}\right)}{U_{1}\left(t,x_{1}\right)}\right)$$

### 2.2. Signal Processing and Displacement Calculated

**Figure 2:**
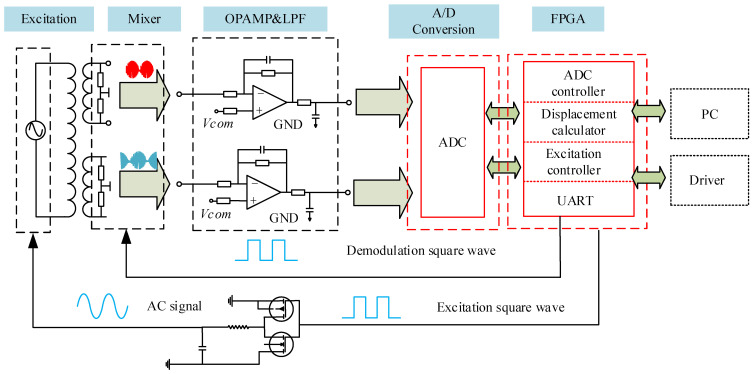
Signal processing diagram of the encoder.

The encoder’s signal processing, based on amplitude discrimination, is illustrated in Figure 2. A 2 MHz square wave is generated by an FPGA chip and driven by a CMOS circuit to excite the coils. According to the measurement principle, this excitation produces two inductive signal channels whose amplitudes are functions of displacement. These signals are subsequently conditioned through a low-pass filter and a power amplifier. The conditioned analog signals are digitized by an ADC module, and the displacement is finally calculated using a dedicated algorithm.

Following the digitization of the inductive signal, an accumulated averaging method is applied to suppress noise. Due to structural non-uniformity between the Ic_Sin and Ic_Cos coils and inherent mismatches in the two signal processing channels, the amplitudes and DC offsets of the two digitized signals differ, which would introduce measurement error. To mitigate this, a data preprocessing module is implemented. First, the signal amplitudes over one encoder pitch are acquired. The DC offsets and the amplitude ratio are then calculated and used for real-time signal compensation. For fast and accurate displacement calculation, the coordinate rotation digital computer (CORDIC) algorithm is implemented on the FPGA [29]. Finally, the computed displacement data is transmitted to a host computer via a serial interface.

## 3. Simulation of Encoder Model

A 3D model of the encoder is developed to verify the structural design and measurement principle. The model is subsequently simulated and validated using the finite element method (FEM), with the simulation parameters detailed in Table 1.

**Figure 3:**
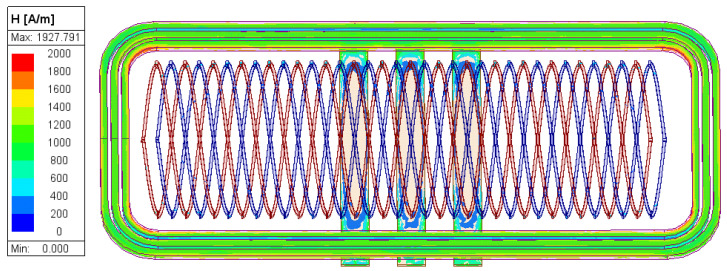
Simulated magnetic field distribution (FEA).

**Figure 4:**
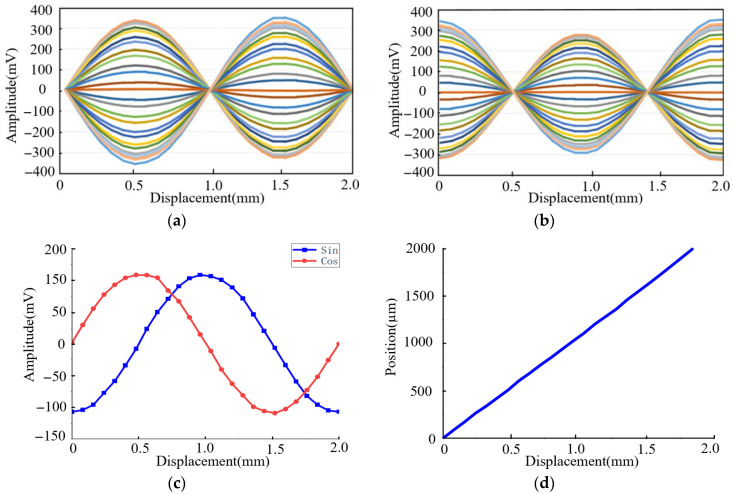

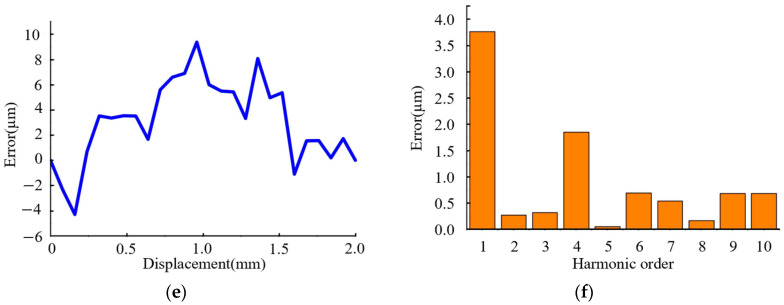
Simulation results: (**a**) simulated inductive signal of Ic_sin; (**b**) simulated inductive signal of Ic_cos; (**c**) amplitude envelope of Ic_sin and Ic_cos at 25 ns; (**d**) calculated displacement; (**e**) calculated vs. configured displacement deviation; (**f**) frequency spectrum of the deviation.

When a 2 MHz, 0.1 A excitation signal is applied to the coils, the resulting magnetic field distribution is shown in Figure 3. The field is symmetric along the x- and y-axes and varies with both time and displacement. Ideally, based on the measurement principle, the amplitude envelopes of the inductive signals should be perfect sine and cosine curves. However, the magnetic field—disturbed by the excitation coils and eddy currents—is not perfectly uniform, introducing harmonic components beyond the fundamental frequency. Additionally, the non-ideal coupling area of the inductive coils further distorts the signal. Consequently, the amplitude envelopes of the inductive signals contain these harmonic components, which introduce measurement errors. The simulated inductive signals are presented in Figure 4a,b, where the x-axis represents the displacement of the movable part, and each curve corresponds to the signal at a discrete time step. Selecting a specific time instant of 25 ns, the amplitude envelopes versus displacement are plotted in Figure 4c, showing a close approximation to sinusoidal and cosinusoidal curves. The displacement calculated using Equations (6) and (7) is plotted in Figure 4d. Compared to the configured motion path, the deviation curve in Figure 4e shows a peak-to-peak error of 13.8 μm within one pitch. A Fast Fourier Transform (FFT) of this deviation, shown in Figure 4f, reveals that the harmonic components are dominated by the first- and fourth-orders. These results confirm the feasibility of the encoder’s structure and measurement principle.

## 4. Experiment

### 4.1. Encoder Prototype and Experiment Platform

**Figure 5:**
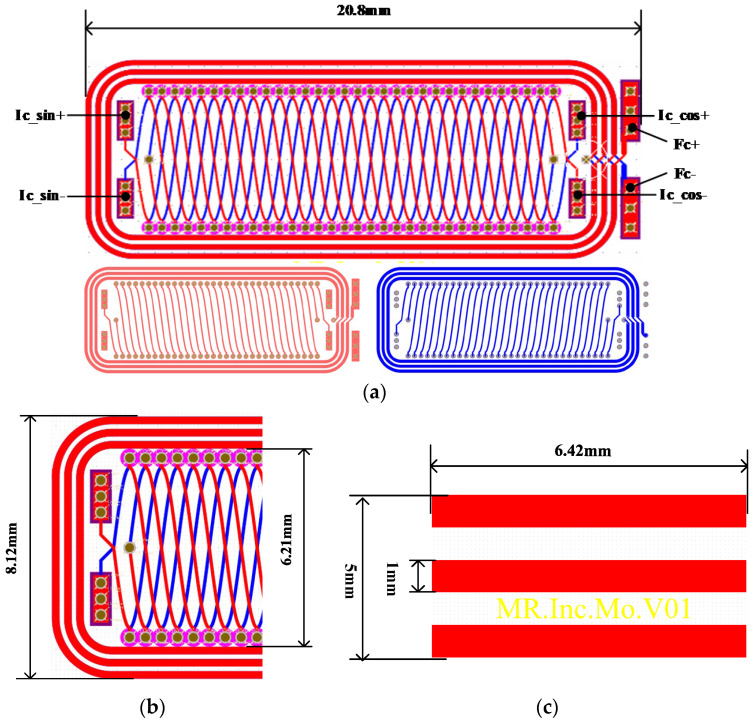
PCB layout of the encoder: (**a**) PCB diagram of the stationary part; (**b**) detailed view of the stationary part; (**c**) PCB diagram of the movable part.

The encoder prototype is fabricated using printed circuit board (PCB) technology. Key manufacturing parameters for the stationary and movable parts are listed in Table 2, with the physical PCB assembly shown in Figure 5. The stationary part is a double-layer PCB with an overall length of 20 mm and an effective measurement stroke of 18 mm. Its top- and bottom- layers are depicted in Figure 5a, where the coils on both layers are interconnected through vias. This part has a width of 9 mm and a plate thickness of 0.4 mm. The sinusoidal (Ic_sin) and cosine (Ic_cos) inductive coils are offset by W_p_/4 along the measurement axis. The movable part, shown in Figure 5b, is a single-layer PCB carrying three metal conductive plates, with dimensions of 6.42 mm × 1 mm and fabricated with 1 oz copper to generate eddy currents.

**Figure 6:**
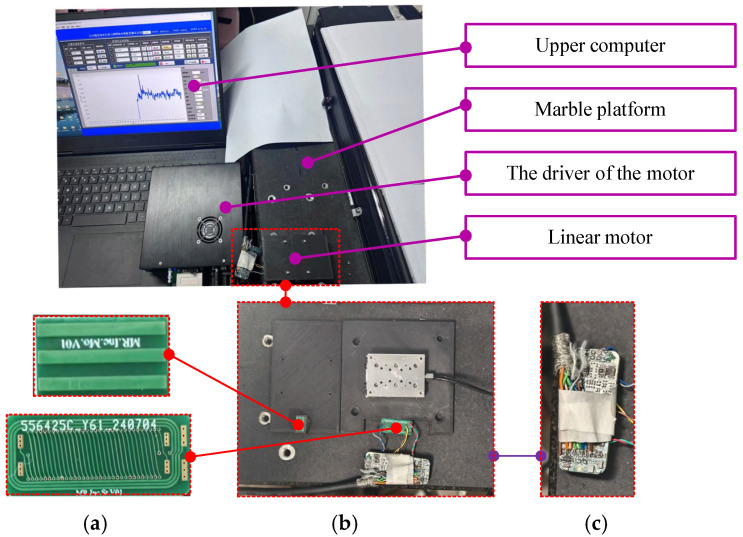
Encoder prototype and experiment platform: (**a**) detailed view of the encoder prototype; (**b**) overall view of sensor prototype, signal processing board, and piezoelectric linear motor; (**c**) detail view of signal processing board.

To evaluate the performance of the fabricated encoder prototype, a dedicated experiment platform is constructed, as illustrated in Figure 6. The platform integrates a high-precision piezoelectric linear motor (with its driver), a marble base, the encoder under test, and a host computer. As shown in Figure 6a, the encoder’s stationary part is mounted on a marble platform with a flatness below 10 μm to ensure stability. The movable part is fixed to the stage of the piezoelectric linear motor (Figure 6b), which provides the reference motion. This motor is equipped with a high-precision optical grating (10 nm resolution, ±1 μm accuracy) and a minimum step size of 0.01 μm, serving as the displacement reference. The signal processing board (Figure 6c) is connected to the planar coils on the stationary part via twisted-pair cables.

### 4.2. Experiment Results

**Figure 7:**
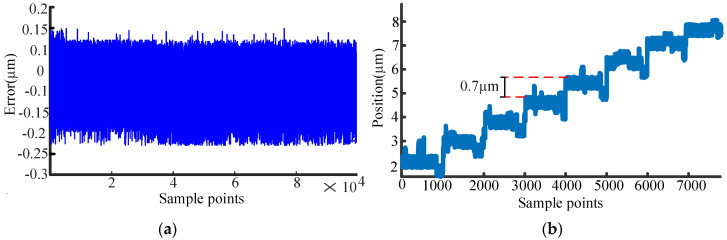
Experiment results: (**a**) experimental results of stability; (**b**) experimental results of resolution.

The stability of the encoder is evaluated first. To ensure measurement reliability, the test is conducted under rigorously controlled environmental conditions, with the temperature maintained at 26 ± 0.5 °C and the relative humidity at 60 ± 1% RH. The movable part of the encoder is kept static throughout the 15 min sampling time. As shown in Figure 7, the experimental results indicate that the encoder achieves a peak-to-peak stability of 0.35 μm.

The resolution, which defines the smallest detectable change in position, is a critical parameter for displacement encoders. The resolution of the prototype is tested by first driving the linear motor with a minimum step size of 0.01 μm. Subsequently, the step size is incrementally increased to identify the threshold at which a discernible variation in the encoder’s output displacement could be confirmed. A clear output corresponding to a 0.7 μm motion step is demonstrated in Figure 7b, establishing this value as the encoder’s resolution.

**Figure 8:**
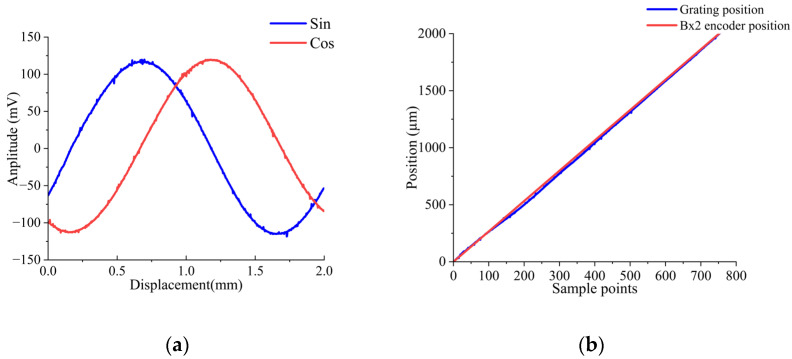
Experiment results: (**a**) Analog voltage output of Ic_Sin and Ic_Cos processed by the Op-Amp and LPF module; (**b**) The comparison diagram of calculated displacement and output displacement of the optical grating.

The analog voltage outputs of Ic_Sin and Ic_Cos, conditioned by the op-amp and LPF circuit during one-pitch movement, are demonstrated in Figure 8a. Both signals exhibit experimental amplitudes of 240 mV, consistent with the simulation results in Figure 3. A comparison between the encoder’s calculated displacement and the grating reference is presented in Figure 8b.

**Figure 9:**
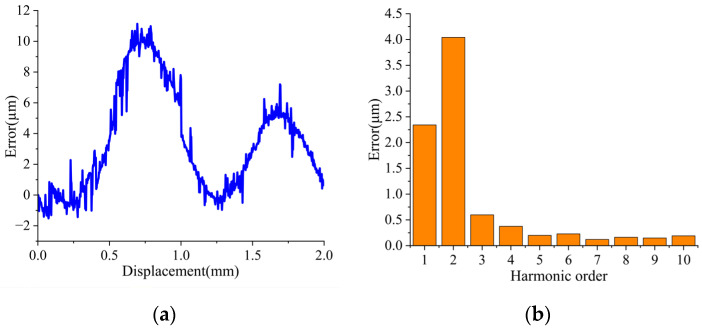
Calibration experiment results: (**a**) measurement error within one pitch; (**b**) frequency spectrum of the measurement error.

A comparison with the optical grating data yields the encoder’s measurement error of the encoder within one pitch, as shown in Figure 9a, where a peak-to-peak error of 12.8 μm is observed. Subsequent FFT analysis, presented in Figure 9b, indicates that the error spectrum is dominated by first- and second-order harmonics. The first-order harmonic is mainly traced to systematic imperfections such as coil installation errors, manufacturing tolerances, and wiring asymmetry. The second-order harmonic stems from deviations in the ideal quadrature relationship between the Ic_sin and Ic_cos signals, often due to phase non-orthogonality or amplitude mismatch. The error profile also contains intermittent glitches, largely attributable to electromagnetic interference (EMI) from the linear motor. Mitigation of these artifacts is achievable through appropriate hardware and software filtering.

The performance comparison of the proposed encoder against previously reported work is presented in Table 3.

## 5. Conclusions

A compact linear inductive encoder featuring a 15 mm measurement range is presented. The work encompasses the introduction of the encoder’s structure and working principle, the construction of a 3D model, and the execution of electromagnetic field simulations to verify design feasibility. Subsequently, a prototype is manufactured via PCB technology and tested on a custom-built experiment platform. Systematic evaluations of stability, resolution, and accuracy are performed. The tests confirmed a peak-to-peak stability of 0.35 μm, a resolution of 0.7 μm, and an accuracy of 12.8 μm. The encoder offers the benefits of compact size, simple structure, and low cost, showcasing considerable application potential.

## Figures and Tables

**Table 1:** Simulation parameters of the encoder.

Parameter	Value	Unit
Pitch (W_p_)	2	mm
Turns of excitation coils	10	turns
Turns of inductive coils	1	turns
Resistance of excitation coils	10	Ω
Resistance of inductive coils	1	MΩ
Frequency of the excitation signal	2	MHz
Amplitude of excitation signal	0.1	A
Linear displacement step	0.08	mm
Simulation time	500	ns
Materials of coils	Copper	
Air gap between the stationary part and the movable part	0.1	mm

**Table 2:** Manufacturing parameters of the sensor prototype.

Parameter	Value of Stationary Part	Value of Movable Part
Number of layers	2	1
Line width of the inductive coils	0.1 mm	N/A
Line width of the excitation coils	0.254 mm	N/A
Clearance between tracks	0.1 mm	N/A
Copper thickness	1 oz	1 oz
Via diameter	0.4 mm	N/A
Volume of the board	22 mm × 10 mm × 0.4 mm	10 mm × 6 mm × 0.4 mm

**Table 3:** Comparison of different linear displacement encoders.

Encoder Schemes	Size (mm)	Range (mm)	Accuracy (μm)	Resolution (μm)	Cost	Sensitive to Environment
Capacitive Time-Grating encoder [16]	N/A	200	4 × 10^−4^	N/A	Expensive	Yes
Capacitive displacement encoder [21]	N/A	15	0.026	9 × 10^−4^	Expensive	Yes
Novel GEC Absolute-Position Encoder [23]	34.3 × 11.4	300	15	N/A	low	No
Encoder with E-Core Dual Planar Coils [24]	100 × 30	70	N/A	2	low	No
Encoder with U-Core Dual Planar Coils [25]	100 × 60	86	N/A	6.5	low	No
Bilateral Sensing Inductive Displacement encoder [26]	400 × 38	288	15	1.2	low	No
This work	20.8 × 8.12	16	12.8	0.7	low	No

## Data Availability

The data that support the findings of this study are included within the article.
